# Grassroots e-floras in the Poaceae: growing GrassBase and GrassWorld

**DOI:** 10.3897/phytokeys.48.7159

**Published:** 2015-04-15

**Authors:** Maria S. Vorontsova, Derek Clayton, Bryan K. Simon

**Affiliations:** 1 Comparative Plant & Fungal Biology, Royal Botanic Gardens, Kew, Richmond, Surrey, TW9 3AE, United Kingdom; 2 Queensland Herbarium, DSITIA, Brisbane Botanic Gardens, Mt Coot-tha, Toowong, Qld 4066, Australia

**Keywords:** Classification, DELTA, grasses, e-taxonomy, Scratchpads

## Abstract

GrassBase and GrassWorld are the largest structured descriptive datasets in plants, publishing descriptions of 11,290 species in the DELTA format. Twenty nine years of data compilation and maintenance have created a dataset which now underpins much of the Poaceae bioinformatics. GrassBase and GrassWorld can continue to grow productively if the proliferation of alternative classifications and datasets can be brought together into a consensus system. If the datasets are reconciled instead of diverging further apart a long term cumulative process can bring knowledge together for great future utility. This paper presents the Poaceae as the first and largest model system for e-taxonomy and the study of classification development in plants. The origin, development, and content of both datasets is described and key contributors are noted. The challenges of alternative classifications, data divergence, collaborative contribution mechanisms, and software are outlined.

## Grasses as the first plant e-taxonomy model system

*Grasses and floristic knowledge.* Grasses (Poaceae) are undisputedly the most economically important family of flowering plants (www.fao.org), including wheat (*Triticum aestivum* L.), rice (*Oryza sativa* L.), maize (*Zea mays* L.), sugar cane (*Saccharum officinarum* L.), bamboos, forage grasses, and lawn grasses. As a group so fundamental to the human civilisation the grasses have always been well studied and the cumulative body of knowledge on the circa 12,000 species (Clayton et al. 2014, Simon et al. 2014) is considerably greater than that available for any other group of plants. The global biodiversity inventory of the grasses at the species-level is advanced compared to other families and Flora treatments are available for Europe (Tutin et al. 1980), Central America (Davidse et al. 1994), North America (Barkworth et al. 2003, 2007), West Africa (Hutchinson and Dalziel 1972), East Africa (Clayton 1970, Clayton et al. 1974, Clayton and Renvoize 1982), and Zambia to Mozambique (Launert 1971, Clayton 1989, Cope 1999, 2002), with completed and ongoing Flora-writing projects in many other parts of the world (for a list see World Grass Floras in Simon et al. 2014). Plant morphology and spikelet structure are largely uniform across the family lending themselves well to structured descriptions. Grasses are a natural model system for the development of bioinformatics and e-taxonomy in plants because of the large body of data available, a real need for organised and accessible data, and a broad community of specialists across the world.

*DELTA as a standardization tool for descriptions.* The idea of storing standardised species descriptions as a database which could be automatically converted to text was first developed by Mike Dallwitz for insects (Dallwitz 1974) and was implemented using a set of standard data formats and programs called the DEscription Language for Taxonomy (DELTA, Dallwitz 1993 onwards). Grasses were chosen as the first model system in application of the new DELTA system to plants, in collaboration with Leslie Watson at the Australian National University (Watson and Dallwitz 1981). The DELTA Grass Genera of the World dataset (Watson and Dallwitz 1992, 1994) was developed concurrently with the DELTA software and with the implementation of DELTA in numerous plant and animal groups (for a list of references see Dallwitz 1993 onwards).

## GrassBase: baseline data compilation

*How GrassBase grew.* Poaceae Flora treatments written at the Kew Herbarium provided the starting material for Derek Clayton to compile the family generic conspectus Genera Graminum (Clayton and Renvoize 1986), changing numerous generic and tribal concepts. The DELTA software platform was chosen to expand Genera Graminum to a species-level taxonomic treatment in a database format to create an infinitely updatable flora with a useful life span far beyond that of a static publication: GrassBase was born. A summary of the aims of GrassBase is presented in Appendix 1. A new DELTA character set independent from Watson and Dallwitz’s work was developed with a particular emphasis on smooth natural language wording of the descriptions, working with Mike Lazarides at CSIRO Canberra supported by a CSIRO grant in 1985. Species descriptions from Floras, monographs, and taxonomic revisions were translated into the new DELTA character set and species names were arranged in line with the Genera Graminum classification. Nomenclature, synonymy, type, and species distribution data were managed in a separate SYNON Access database. It is estimated that between 1985 and 2014 Derek Clayton spent approximately fifteen hours a week on data entry and management. As GrassBase grew to become the largest DELTA dataset it became too cumbersome for manual editing and additional scripts were written to automate more of the data management tasks. A set of Visual Basic programs called GrassUtils were written by Derek to create automatic links between the DELTA dataset and the Access database; scripts were created by Kehan Harman to produce a single web page description for every species and every genus of the Poaceae (Harman 2007; Harman and Clayton 2007; Clayton et al. 2014). Custom scripts continue to be adjusted for the efficient production of web pages by Nick Black at RBG Kew.

*GrassBase content.* The March 2014 release of GrassBase includes 64,213 Poaceae names published at species rank or below, descriptions of 11,313 accepted species, and 713 genera (Clayton et al. 2014). Nomenclature, synonymy, type, and species distribution data are available for download as part of the SYNON Access database. The SYNON database includes a range of macros, queries and list making tools to aid floristic work, such as a query to generate a list of regional endemic species for any TDWG region. Error checking queries to ensure congruency between data tables are included. A description of every accepted species is available as text automatically translated from the DELTA code on a separate webpage with a stable URL (species descriptions vary in the level of detail depending on how much information was available in the source publication). Generic descriptions are obtained by combining the species descriptions using the DELTA ‘gesumm’ program and the description of every accepted genus is also available as text on a separate webpage. The species-level dataset is available in the format for the interactive key and data querying program INTKEY (Dallwitz 1993 onwards). The generic level dataset is not available in INTKEY format as this was not judged to be a reliable means of specimen identification to genus. The DELTA ITEMS dataset is available on request following acceptance of a data supply agreement.

*GrassBase maintenance.* New Poaceae names recorded by the International Plant Names Index (IPNI 2014) are downloaded every six months and added to the SYNON database. Descriptions of accepted species are translated into the DELTA format using the 1,090 character set. User-contributed amendments and additions are accumulated in the working copies of both name and description datasets, which may include changes to the character set. The two data sets are reconciled every six months using custom scripts, a new Access database is published, new INTKEY files are made, and a fresh set of web pages is generated, a process which takes circa two working days. More detail on the system specification is available in Harman (2007), Harman and Clayton (2007), and on the website.

*Holes in GrassBase.* The original compilation of GrassBase was an ambitious pioneering project aiming to demonstrate the feasibility and usefulness of an electronic flora in comparison to a printed book. Timely project completion was a priority and much was inevitably omitted from the database design. Descriptions lack source attributions and no references are provided for the synonymy. Hybrids are not included. The original set of names in GrassBase was taken from IPNI which did not list infraspecific taxa prior to 1970; the focus of GrassBase remains on the species-level so numerous subspecies and varieties are not included except as synonyms of their species. When a new species is published in a genus not accepted by GrassBase it is moved to the GrassBase genus under a provisional unpublished new combination to maintain the consistency of generic concepts across the database, a process which has created approximately 150 accepted species names which do not correspond to current usage and have not been validly published. There are no plans to publish these names and they have been omitted from all derivative data sets. Every new name recorded by IPNI is reviewed by Derek Clayton before incorporation into GrassBase, but publications with changes to synonymy which do not publish a nomenclatural novelty are unfortunately not always noted. The consistency of the dataset on the global scale reflects the available knowledge and literature which is far from uniform, both in terms of the information provided by individual descriptions and the coverage and age of treatments for different parts of the world.

## GrassWorld: growing beyond the baseline

*Grasses in many languages, reclassified, with pictures.* The GrassWorld project was started by Bryan K. Simon in 2003 to build on GrassBase and gather together all information on the world’s grasses within the DELTA system, to enable the user to query any data type via INTKEY (Simon 2007). The DELTA dataset was built by Bryan Simon with Daniel Healy and Yucely Alfonso, following on from their popular taxonomic resources for the grasses of Australia: AusGrass (Sharp and Simon 2002) and AusGrass2 (Simon and Alfonso 2014). An average of three days per week has been dedicated to the project by the three people since 2006. A decision was taken to follow phylogenetically circumscribed genera to link modern research data to historic literature. Somewhat narrower species concepts were also used, increasing the number of accepted species from 11,313 in GrassBase to 12,100 in GrassWorld. All basionyms and some recent synonyms were added to DELTA. The GrassWorld system now provides species distribution maps, phylogenetic trees, references to published illustrations, and images. The literature section presents an extensive ENDNOTE database. A section dedicated to agrostologists includes CVs, links, and copies of obituaries. The DELTA character set has been translated into three languages following similar work by Watson and Dallwitz: German (by Philip Sharpe, Queensland herbarium, and Hildemar Scholz, B), French (by Philipe Morat, P) and Spanish (by Gilberto Ocampo, CAS) with descriptions available for download in pdf format.

*Grasses in a Scratchpad.* The development of the collaborative platform Scratchpads (Smith et al. 2009) provided an opportunity to present GrassWorld data online (Simon et al. 2014). On 1 January 2014, the GrassWorld Scratchpad was ranked third among the Scatchpads viewed globally (925,608 views) and AusGrass2 was fourth (685,095 views); they were also the first and second most viewed plant Scratchpads. The transfer of GrassWorld data to the Scratchpad environment was carried out through the assistance of Kehan Harman and Irina Brake (Natural History Museum), with data work by Daniel Healy and Yucely Alfonso. Transfer of data to Scratchpads 2 is being carried with the assistance of Dimitris Koureas (Natural History Museum) and Isa Vandevelde (Natural Sciences Museum, Belgium).

*GrassWorld development.* GrassWorld continues to grow as new data is imported from GrassBase and published literature and online resources are added. GrassWorld and part of AusGrass2 have been supported by Bryan Simon in the absence of project funding and unfortunately the development of GrassWorld in its present form will not continue beyond ca 2020. A future merger of the GrassBase and GrassWorld data may be the best option for preserving data.

## Growing apart: the divergence of classifications and datasets

*Divergence of grass classifications.* The purpose of GrassBase was originally defined as a “practical catalogue of identifiable taxa, stable and conservative, a flora which the database seeks to emulate”, in contrast to “a phylogeny according to the latest theory; volatile and not always practical”. The GrassBase classification follows Genera Graminum (1986) with minor amendments and the Poaceae herbarium sequence at RBG Kew reflects GrassBase via a curation policy known as “curation based taxonomy”. GrassBase is the only currently used Poaceae classification which does not follow the now well established sequence of subfamilies and tribes published by the Grass Phylogeny Working Group system (Grass Phylogeny Working Group 2001, Grass Phylogeny Working Group II 2012). In contrast GrassWorld reflects research data broadly in agreement with the two modern generic level reference treatments: the Catalogue of New World Grasses (Soreng et al. 2014) and Kellogg (in press). The generic level reference treatment published by the Catalogue of New World Grasses presents a phylogenetic classification independently compiled from literature and research data by Robert Soreng at the Smithsonian Institution. The first phylogenetic monograph of the family is currently in press; it documents a sequence of all known clades and their synapomorphies, descriptions of genera and clades of generic rank with an emphasis on synapomorphies, keys to all genera, and a synthesis of multidisciplinary research results relevant to the Poaceae evolution (Kellogg in press).

*How far have GrassBase and GrassWorld diverged?* An estimated 10% of species in GrassBase have generic placements different from those in GrassWorld, Catalogue of New World Grasses and the forthcoming Kellogg (in press) treatment. The rearrangement of the classification system according to phylogenetic research results has led to 10% of the species names being reassigned to a different genus, a figure which could rise to 20% when species sampling for molecular studies approaches completeness (Vorontsova and Simon 2012).

*Divergence of other name databases.* Edited versions of GrassBase data contribute to the complexity of taxonomic datasets in the grasses. A copy of the GrassBase SYNON Access database name data made in 2006 has provided the Poaceae data for the World Checklist of Selected Plant Families (WCSP 2014). The edited WCSP Poaceae dataset is now used by The Plant List (2013) and by eMonocot (2014); these are broadly congruent with GrassBase although not identical. It should be noted that the overall global online dataset divergence is considerably more complex than described here when a plethora of other datasets are taken into consideration, e.g. Euro+Med (2006-) which uses more narrow species concepts for European Poaceae.

## Challenges and opportunities

*Can divergent datasets become a consensus classification?* The curator of each database decides which names are accepted. With some 12,000 species the Poaceae are too large for one person to hold in-depth knowledge across the family: in GrassBase a considerable part of the decision making is carried out by data curators who are not taxonomic group specialists. The work of scanning new publications and making decisions on the accepted names is carried out by a different person for each database, sometimes producing a confusing diversity of taxonomic opinions. Considerable resources are spent by users trying to decide which classification to adopt and which names are correct. Developing a process of working towards a single consensus opinion could bring benefits: direct changes to the classification by taxon specialists, as well as time saving for data curators and for users. Database maintenance and the translation of new species descriptions into DELTA format are time consuming tasks which could be distributed between different people.

*How do we collaborate towards a consensus?* Compilers of any dataset cannot fail to introduce unintentional biases reflecting their areas of expertise. Regional floristic specialists are commonly in disagreement with taxonomic group specialists regarding species delimitation. Taxonomists in biodiversity-rich countries can lack adequate internet connections to view the outputs of e-taxonomy, let alone participate. Engaging the full range of people who can provide useful information for species-level descriptions is challenging. Considerable resources are needed to incorporate published information into an electronic dataset. Data contributors should be fully acknowledged and have ownership of their contributions, while data quality and consistency still needs to be maintained across the dataset. The area of collaborative e-taxonomy is still in development.

*Lack of consensus at species-level?* The global community is now broadly in agreement regarding subfamilies and tribes of the Poaceae (Grass Phylogeny Working Group II 2012, Soreng et al. 2014, Kellogg in press). It is possible that a consensus generic classification will emerge once the species-level sampling in phylogenetic studies is more complete. The variation in species concepts is such, however, that a species-level consensus may never be reached. Simultaneous alternative interpretations and concepts are currently not accommodated by any existing taxonomic data system although it is possible that this could be developed.

*Grass Genera of the World: data incompatibility challenge.* While alternative classifications are a frequent focus of debate, divergent and incompatible datasets are arguably a greater concern when viewed in the context of long term information accrual. The Grass Genera of the World DELTA dataset (Watson and Dallwitz 1992, 1994) was designed as a compendium of information on grasses to aid and inspire research and includes a broad range of information not addressed by GrassBase or GrassWorld: anatomical characters, photosynthetic pathways, pathogen specificity, and economic uses. The dataset was compiled from literature as well as derived from original specimen observations and associated research projects (e.g. Macfarlane 1979, Webster 1987, van den Borre and Watson 1997) and grew to be an influential body of reference material on the grasses. Grass Genera of the World data is recorded for every genus while many of the generic concepts have changed: integrating this information with species-level flora descriptions collected by GrassBase would be challenging. A range of other DELTA datasets in the grasses (listed by Dallwitz 1993 onwards) hold valuable information that could be integrated: species-level data developed by Les Watson in Australia in collaboration with taxonomic specialists, including the Paniceae, Chloridoideae, Pooideae, *Enneapogon*, *Digitaria*, *Sporobolus*, and *Aristida*; grasses of southern Africa (Gibbs Russell et al. 1990); New World Paniceae datasets developed by Webster (e.g. Webster and Valdés Reyna 1988; Webster et al. 1989).

*Bamboos in GrassBase.* The GrassBase character set was designed with specimen identification as a primary consideration, and some morphological terminology specific to the bamboos was altered for compatibility with non-bamboo grasses, with advice from Christopher M. A. Stapleton. This has enabled the use of INTKEY to distinguish between bamboos and other grasses, but has created discrepancies between terminology in GrassBase and that used in bamboo specialist literature (e.g. Clark 2014). The implementation of technically correct terminology would improve the usefulness of GrassBase to bamboo specialists but would also create an incompatible subset of bamboo descriptions within the dataset.

## e-Infrastructure for grasses

*The software challenge.* The DELTA software suite has been in development for over 30 years and lacks full functionality under 64-bit Windows (Baird 2010). Alternatives to the original software are now becoming available from the Atlas of Living Australia DELTA written in Java (Open Delta 2014) and Free DELTA (2014) although neither of these have been able to fully replace DELTA to date. Open Delta software accommodates most data entry tasks and manipulation of ITEMS files; Open Delta CHECK successfully identifies erroneous character states but fails to identify errors in character dependency. A full update to GrassBase using Open Delta may be possible following further development although testing in early 2014 has demonstrated this is not possible at the time of writing. GrassUtils and other custom scripts will need considerable redevelopment. An alternative strategy could look at novel descriptive data formats outside the DELTA system.

*The web integration challenge.* Multiple contributor e-taxonomy websites, where multiple users in different countries are able to edit the same website simultaneously, publish descriptions as plain text. DELTA datasets are translated into text prior to web publication and the reverse process of obtaining DELTA code from text descriptions is currently not possible. The DELTA system lacks multiplatform interoperability while LUCID (2014) lacks the full functionality of the DELTA software suite. A future multi-contributor web based data system could not integrate with DELTA datasets online without further software development.

*Growing an e-flora into a multipurpose e-infrastructure platform.* The original name for GrassBase was “World Grass Flora” to reflect its design as a database equivalent of a traditional flora: a species inventory and an identification guide. GrassWorld has started to expand the range of information available. If these resources are to integrate with the modern eBiosphere online and contribute effectively to the World Flora Online (Global Strategy for Plant Conservation, http://www.plants2020.net) a radical modernisation of web presentation will be necessary, including links to observation data, plant ontogenies, and provision of machine readable data output.

## Conclusions

This paper argues that a collaborative approach and careful thought across the Poaceae taxonomic community are needed to take grass e-taxonomy forwards. Failure to plan and collaborate could lead to an increasing proliferation of contradictory classifications. Unique datasets of great value could be rendered obsolete if software development and database maintenance does not keep pace with technology platforms. Investment in community database integration and infrastructure could unlock untapped research and data mining potential of many historic datasets. The rich data and the long history of database compilation in the grasses present an unprecedented opportunity to study the development of classifications and to develop e-taxonomic models. The authors would like to invite potential collaborators to discuss dataset improvements and future plans.

## Appendix 1

**Aims of GrassBase as summarised by Derek Clayton in 2012.**

Grassbase is a natural extension of Genera Graminum and the big Regional Floras produced at Kew, expanding them to a global treatment at species-level, and exploring the concept of a continuously updated e-Flora for a family of some 11000 species. Its design is based, firstly, on defining its intended applications; secondly on identifying all the retrieval and exploratory tasks commonly undertaken in the course of these applications, then building the database to service these tasks. Applications and tasks are outlined below:

1. As a public Flora. Floras are the essential reference manuals underpinning all plant science. Grassbase emulates their traditional content, embracing nomenclature, description, identification and distribution.
  **Tasks.** Display the classification currently adopted at Kew. Identify specimens. Clarify nomenclature. Investigate plant geography.
2. As a source for expediting the production of local Floras or Field Keys for public use.
  **Tasks.** List the local flora. Deliver chunks of text for pasting and editing on an external file. Write keys.
3. As a workspace and toolkit for research on the family’s morphological classification from species to tribal level. It involves detection and resolution of inconsistencies in nomenclature, identity or relationships, and reconciliation with external datasets such as DNA.
  **Tasks.** Enable the user to rummage for relevant information throughout the system seeking solutions to problems. Assist comprehension of the overall classification. Support the hatching and testing of innovative ideas.
